# Detection of Gas Drifting Near the Ground by Drone Hovering Over: Using Airflow Generated by Two Connected Quadcopters †

**DOI:** 10.3390/s20051397

**Published:** 2020-03-04

**Authors:** Ryohei Sato, Kento Tanaka, Hanako Ishida, Saki Koguchi, Jane Pauline Ramos Ramirez, Haruka Matsukura, Hiroshi Ishida

**Affiliations:** 1 Graduate School of Bio-Applications and Systems Engineering, Tokyo University of Agriculture and Technology, Tokyo 184-8588, Japan; satoryohei914@gmail.com (R.S.); tana.ken.smash77@gmail.com (K.T.); hanako.ishida05@gmail.com (H.I.); sakitat.190326@gmail.com (S.K.); h_ishida@cc.tuat.ac.jp (H.I.); 2 Research Fellow of Japan Society for the Promotion of Science, Tokyo 102-0083, Japan; 3 Department of Mechanical Engineering, University of Philippines Diliman, Quezon City 1101, Philippines; jane.prramirez@gmail.com; 4 Graduate School of Engineering Science, Osaka University, Osaka 560-8531, Japan

**Keywords:** gas source localization, drone, multicopter, gas sensor, impinging jet

## Abstract

This paper describes the utilization of the downwashes of multicopters for gas-sensing applications. Multirotor drones are an attractive platform for sensing applications. Their high maneuverability enables swift scanning of a target area with onboard sensors. When equipped with a gas sensor and used for gas-sensing applications, however, the strong downwash produced by the rotors poses a problem. When a multicopter is hovering at a low altitude, gas puffs leaked from a gas source on the ground are all blown away. Here, we propose to use two multicopters connected by a rod or a string and place a gas sensor at the midpoint of the rod/string. The downwash generated by each multicopter spreads radially after it impinges on the ground. When two multicopters are connected, the airflows spreading radially along the ground from the two multicopters impinge at the center and are deflected in the upward direction. Gas puffs wafting near the ground surface between the two multicopters are carried by this upward airflow to the gas sensor. Experimental results are presented to show the soundness of the proposed method. The connected quadcopters hovering over an ethanol gas source was able to detect the gas even with a moderate cross-flow.

## 1. Introduction

Mobile robots can be used as moving platforms for various sensing tasks. Robots can carry various sensors and autonomously collect sensor data even in hostile environments where the risk is too great to dispatch human workers. When equipped with gas-sensing devices, robots can be used for localizing hazardous gas leaks and mapping chemically contaminated areas [1,2]. Such gas-sensing robots can be also applied to environmental monitoring [3]. For example, various gases are produced in landfill sites through biological decomposition of organic waste. Monitoring the emission rates of these landfill gases is of great importance because some gases, e.g., carbon dioxide and methane, have greenhouse effects [4,5]. In order to accurately estimate the amount of gas emission from a whole landfill site, we need to measure the concentrations of landfill gases at a number of locations in the landfill site. A robot can autonomously conduct such tedious repetitive measurements.

Therefore, various gas-sensing mobile robots have been reported over the past decades [1,2]. Although most of the robots reported so far have been wheel-based, small unmanned aerial vehicles (UAVs) are an attractive platform for gas-sensing tasks. Unlike wheeled or legged ground-based robots, UAVs can easily move over rough and/or wet terrains. Among various types of UAVs, multirotor drones (multicopters) are most attractive. Winged airplanes [6] and blimps [7,8] have also been applied to gas-sensing tasks. To obtain sufficient lift, however, winged airplanes need to maintain certain flight speeds and blimps need large balloons. The speeds of winged airplanes are often too high for gas-sensing tasks because the response time of typical gas-sensing devices is on the order of several seconds. Blimps lack agility because of their large bodies and inertia. Multicopters, on the other hand, have high maneuverability and are easy to control [9].

In recent years, an increasing number of papers have been published on the use of multicopters for gas-sensing tasks. Gas detection on a UAV platform alone is challenging. If a gas release occurs at the ground level, the concentration of the gas would be significantly diluted when it reaches the UAV flying at a high altitude. However, even an affordable metal oxide gas sensor showed some response when a hexacopter was made to fly at a relatively low altitude over a gas source [10]. A hexacopter equipped with a laser-based remote methane detector [11] was able to detect methane emission from the ground even from 25 m above a landfill site [12]. A gas distribution map can be obtained by scanning the area of interest with a multicopter equipped with such a gas detection device [10,12,13]. Neumann et al. reported results of gas source localization experiments using a quadcopter equipped with several different types of gas sensors (metal oxide, electrochemical, catalytic, and infrared sensors) [14]. In their experiments, the quadcopter was able to track a gas plume over 15 m in an outdoor environment. The location of the gas source was successfully estimated by applying a particle filter-based algorithm to the sensor data collected during the flight of the quadcopter. Successful gas source localization demonstrations were also reported for a quadcopter equipped with a laser-based remote methane detector [15]. Demonstrations of gas distribution mapping and gas source localization using multicopters have also been given for indoor environments [16,17].

Despite these successful examples, multicopters have a major drawback when used for gas-sensing tasks. Their rotors produce a strong downwash to obtain a lift force. Most gas sensors show a response only when a gaseous chemical substance actually touches the sensor surface. The gas contained in the surrounding air needs to be transported to the sensor surface by advection and/or diffusion. When a multicopter flies, its rotors generate strong airflow in the downward direction, which disturbs the spatial gas distribution and affects the gas transport. This issue has been raised even in very early work on gas-sensing drones [9,14]. It is intuitively expected that the gas hanging over a multicopter is sucked into the rotors while the gas drifting underneath the multicopter is blown away. Supporting results were obtained by conducting computational fluid dynamics (CFD) simulations of the airflow field around a multicopter [18,19] and flow visualization experiments [17].

**Figure 1:**
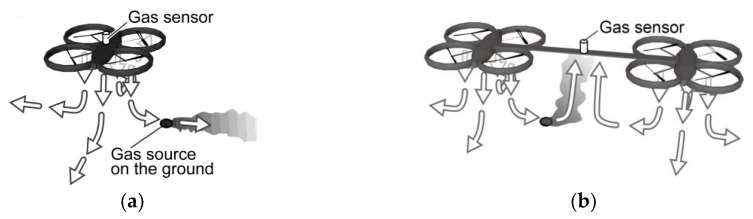
(**a**) Schematic diagram of the air currents generated by a single quadcopter; the gas released from the ground is blown away from the quadcopter. (**b**) Schematic diagram of the air currents generated by two connected quadcopters; the gas released from the ground source is brought to the sensor by the upward fountain flow.

In most scenarios expected for gas-sensing drones, e.g., monitoring of landfill gas emission, the target gas is released from somewhere underneath the drones. Therefore, a gas sensor or a laser-based remote methane detector has often been mounted under the main body of a multicopter [10,12,13]. In some work, a gas sensor was attached underneath a rotor because that is the place to which the gas hanging around the multicopter is collected [9,14,16]. Suppose that the multicopter is flying in a gas plume whose width and height are much larger than the size of the multicopter. The gas sensor would show some response regardless of its position because the multicopter is completely surrounded by the gas. However, the downwash causes a significant impact on the gas sensor response when the multicopter is flying at a low altitude over a small gas source, as shown in Figure 1a.

When a multicopter is hovering near the ground, the airflow blown down from the multicopter impinges on the ground. This type of flow is called an impinging jet, and has been extensively studied in fluid mechanics because of its industrial importance, e.g., in cooling and washing [20]. After the impingement, the airflow spreads radially along the ground, as shown in Figure 1a. This flow is called a wall jet [20]. If a gas source exists in the reach of this wall jet, most of the gas released from the source is blown away from the multicopter. Therefore, the gas sensor mounted on the multicopter will show almost no response. This issue is mitigated when a laser-based remote methane detector is used. The gas drifting near the ground surface can be detected remotely while making the multicopter fly at a high altitude. However, the signal-to-noise ratio degrades when measuring over a large distance. Moreover, there are gases that cannot be remotely detected using the laser-based technique.

In this paper, we propose to exploit the problematic downwash for gas sensing. If two multicopters are connected by a rod or a string, as shown in Figure 1b, the wall jet generated along the ground by one of the multicopters impinges with the wall jet from the other multicopter. Then, the airflow is deflected in the upward direction, and so-called “fountain” flow is created [20,21,22]. If a gas sensor is attached at the midpoint of the rod/string, this upward airflow brings gas puffs wafting near the ground surface to the gas sensor in midair. This type of fountain flow is generated during takeoff and landing of short-take-off/vertical landing (STOVL) aircraft. Studies have been conducted to avoid the fountain flow from affecting the stable flight of STOVL aircraft [21]. Multiple impinging jets are also often used in cooling systems because the heat transfer from an object surface is promoted by multiple jet impingement [23]. In this work, we utilize twin jets to promote mass transport from the ground surface to the gas sensor.

The rest of the paper is organized as follows. In Section 2, the setup of the connected multicopters is described together with the results of CFD simulation of the flow field generated by them. To show the proof of concept, we used two commercially available quadcopters in the experiments and manually controlled their positions in an indoor environment. In Section 3, results of gas detection experiments using a photo-ionization gas detector (PID) are presented. Metal oxide gas sensors have been most widely used in gas-sensing mobile robots and drones because of their high sensitivity, excellent long-term stability, and fair response time [2]. However, the response of metal oxide sensors varies when they are exposed to strong airflow. Therefore, we used a PID in the first set of experiments. We fixed the PID on a tripod and recorded its readings to show that gas puffs drifting near the floor can be brought to an elevated height by using the fountain flow. After checking the response of metal oxide gas sensors to airflow, flight experiments were conducted using two manually controlled quadcopters and a metal oxide gas sensor. The results of this set of experiments are presented in Section 4. It has been shown that the connected quadcopters hovering at a height of 0.5 m over a gas source can detect the gas even with a moderate cross-flow. Finally, conclusions are given in Section 5. This paper is based on our previous conference paper [24], in which the initial results of the flight experiments were presented. The results of the CFD simulation and the flight experiments in a cross-flow are newly added in this paper.

## 2. Materials and Methods

### 2.1. Connected Quadcopters

**Figure 2:**
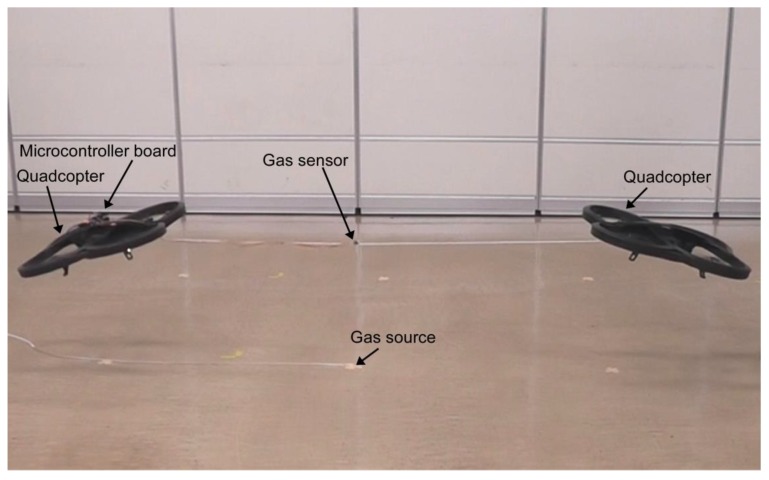
Quadcopters flying over a gas source. Two quadcopters were connected using a polypropylene string. A metal oxide gas sensor was attached at the midpoint of the string. A plastic tube was fixed on the floor using adhesive tape, and ethanol vapor was released from its tip.

In order to experimentally prove the concept of the proposed gas detection method, we used commercially available quadcopters (AR.Drone 2.0 Power Edition, Parrot SA, Paris, France). Their size is 52 cm in length, 52 cm in width, and 11 cm in height. The distance between the two diagonal rotors is 40 cm. In the experiments presented in this paper, each quadcopter was individually and manually controlled by a different experimenter using their smartphone. The distance between the two quadcopters was set to 1.7 m by connecting them using a polypropylene string, as shown in Figure 2. Stronger fountain flow will be generated if we make the distance shorter. However, the flight of the two manually controlled quadcopters will become unstable if they get too close to each other; 1.7 m was the minimum distance that needed to be kept for stable hovering of the quadcopters. When we connected the quadcopters using a rigid rod, we were not able to achieve stable flight. The altitude of the quadcopters was kept at 0.5 m during the experiments. Stronger fountain flow will be obtained if the altitude is decreased. However, 0.5 m was, again, the minimum altitude that needed to be kept to achieve stable hovering.

As shown in Figure 2, a metal oxide gas sensor was attached on the midpoint of the polypropylene string. The sensor was wired to an Arduino Uno microcontroller board (Arduino, Turin, Italy), which was mounted on the left quadcopter in Figure 2. The resistance values of the gas sensor measured by the microcontroller at 10 samples/s were wirelessly transmitted to a laptop computer for recording. The sensor response is defined as the ratio of the resistance value when exposed to a gaseous chemical compound to that in clean air. The value of the sensor response is unity when there is no gas in air, and decreases with gas concentration. It should be noted that the quadcopters shown in Figure 2 are slightly tilted because they were pulling with each other to keep their positions. We can achieve stable level flight of the two connected quadcopters if an autonomous flight control function is implemented. However, the tilt might have had beneficial impacts in the experiments. If the two impinging jets are slightly titled to each other, the fountain flow becomes stronger. It would be interesting to investigate in future the optimum tilt angle.

**Figure 3:**
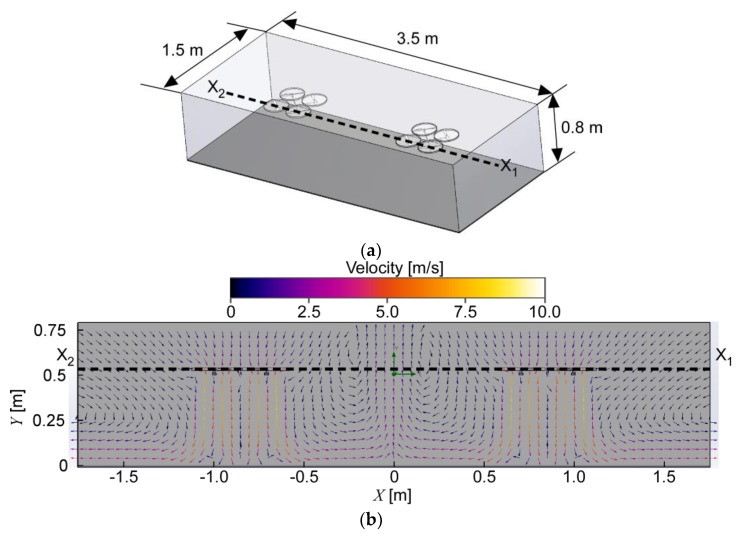
Result of computational fluid dynamics (CFD) simulation. (**a**) Computational domain prepared for simulating the airflow generated by the connected quadcopters; (**b**) velocity distribution in a vertical cross-section that intersects the quadcopters at line segment X_1_X_2_.

Figure 3 shows the result of the CFD simulation obtained using a commercially available CFD software package, SOLIDWORKS Flow Simulation (Dassault Systèmes, Vélizy-Villacoublay, France). The connected quadcopters were assumed to be hovering at 0.5 m from the floor. The steady-state turbulent air currents generated by the rotors were calculated using the *k*–*ε* turbulence model. All rotors were assumed to be driven at the same speed of 3800 rpm, which was determined by measuring the rotational speed of the propeller blades. The diameter and pitch of the propeller were set to 254 and 114 mm, respectively. The computational domain with the size of 3.5 m × 1.5 m × 0.8 m was discretized into 2,201,346 cells. The no-slip boundary conditions were applied to the surfaces of the floor and quadcopters. The open boundary conditions were applied to the side and top walls of the calculation domain so that air could freely move through these boundaries. Generation of the fountain flow is clearly seen in Figure 3. A radial wall jet is generated when the downwash from each quadcopter impinges on the floor. The upward air current is generated at the center of the computational domain at which the wall jets from left and right impinge against each other. The velocity of the wall jet going outward along the floor from one of the quadcopters was measured to be 2.37–2.75 m/s by placing an ultrasonic anemometer (Model 81000, R. M. Young Co., Traverse City, MI, USA) 1 m from the quadcopter. The simulation result was in good agreement with the measured velocity.

### 2.2. Gas Detection Experiments

In this first set of experiments, we used a PID (ppbRAE 3000, RAE Systems, Inc., Sunnyvale, CA, USA) to see if gas puffs wafting near the ground could actually be brought to an elevated height with the help of the fountain flow. Metal oxide gas sensors have been widely used in mobile robot olfaction research because of their high sensitivity to volatile organic compounds, excellent long-term stability over years, and relatively fast response time on the order of several seconds [2]. However, care should be taken because their response varies when exposed to strong airflow. A metal oxide gas sensor has a heater in its metal can package. When in operation, the sensing element of the sensor is heated up to 150–400 °C [25]. When the sensor is exposed to airflow, heat is removed by forced convection. The change in the temperature of the sensing element causes variation in the sensor resistance. On the other hand, the reading of a PID is not affected by airflow. An air sample is sucked into a small ionization chamber in a PID at a constant flow rate using a pump [26]. Gas species contained in the air sample are ionized by shedding ultraviolet light and detected at the electrodes in the chamber.

As the PID we used was too bulky and heavy to put on the quadcopters, we fixed the PID on a tripod. The gas inlet of the PID was placed 0.4 m from the floor in the fountain flow generated by the two quadcopters. In the experiments, each quadcopter was manually controlled to hover at a constant height of 0.5 m over a specified position. However, the quadcopters sometimes lost control for a short period of time and temporarily left the specified positions. The 0.1 m difference in height between the quadcopters and the PID was set to avoid the quadcopters from colliding against the PID in such cases. All experiments were carried out in a closed room with all air-conditioning and ventilation systems turned off. The velocity of the background airflow in the room that was caused by thermal convection was less than 0.03 m/s when measured by the ultrasonic anemometer. Saturated ethanol vapor was released at a constant flow rate of 0.5 L/min from a plastic tube with an inner diameter of 2 mm. The position of the tube was changed to investigate the range of gas detection.

### 2.3. Flight Experiments

**Figure 4:**
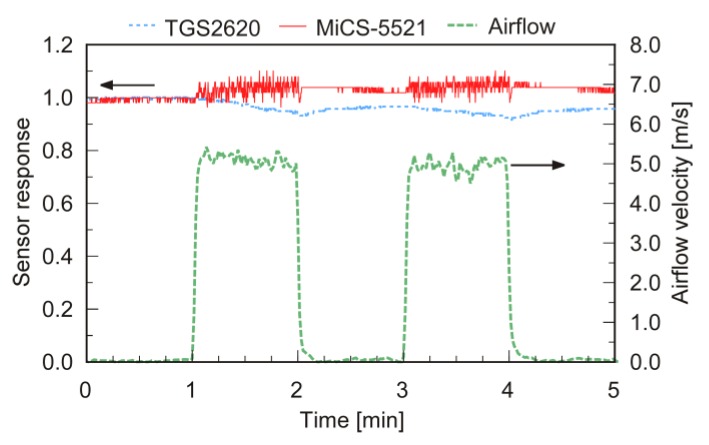
Changes in the response values of two metal oxide gas sensors against airflow. The two gas sensors were placed 0.5 m from an electric fan and exposed to airflow of 5 m/s.

Although metal oxide gas sensors are less stable than PIDs, the lightweight sensors can be easily accommodated on a drone. The second set of experiments were conducted to confirm that gas puffs transported by the fountain flow were detected even when a metal oxide gas sensor was used. Figure 4 shows the result of a preliminary experiment conducted to choose a sensor to place on the connected drones. Two different metal oxide gas sensors, TGS 2620 from Figaro Engineering Inc., Minoh, Japan, and MiCS-5521 from e2v technologies Ltd., Chelmsford, United Kingdom, were placed side by side 0.5 m from an electric fan together with a hot-wire anemometer (CLIMOMASTER MODEL 6541-21, Kanomax Corporation Ltd., Suita, Japan). Changes in the response values of these two gas sensors are shown in Figure 4 with the velocity of airflow simultaneously measured using the anemometer. The gas sensors were repeatedly exposed to airflow of 5 m/s by turning on and off the fan. When the fan was turned on, the response value of the TGS2620 sensor slightly decreased as if responding to gas. On the contrary, the response of the MiCS-5521 sensor showed no significant decrease or increase, although the random noise increased when exposed to the turbulent airflow generated by the fan. Based on this result, we chose the MiCS-5521 sensor and fixed it on the polypropylene string of the connected quadcopters.

The flight experiments using a MiCS-5521 sensor were also carried out in the closed room with all air-conditioning and ventilation systems turned off. Saturated ethanol vapor was released at a constant flow rate of 0.5 L/min from the same plastic tube. In practical applications, e.g., methane emission monitoring in a landfill site, the connected multicopters will almost always encounter a cross-flow. Therefore, seven electric fans were aligned in the room to generate a uniform cross-flow of 2 m/s.

## 3. Results of Gas Detection Experiments

### 3.1. Gas Detection at an Elevated Height

**Figure 5:**
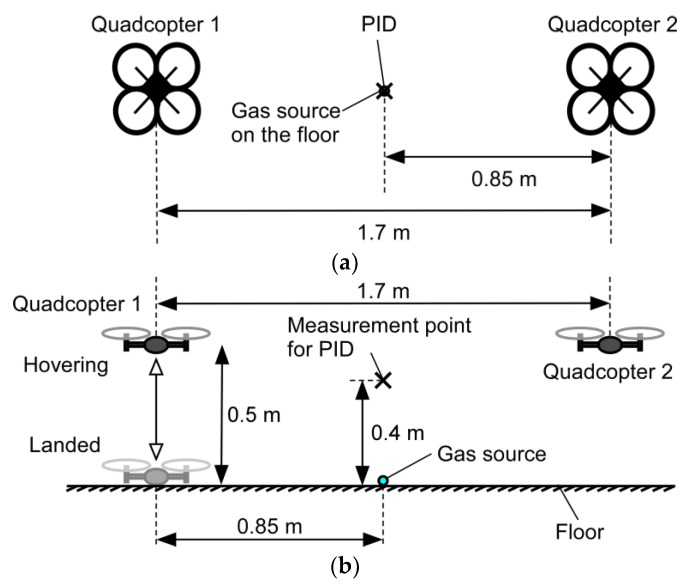
Experimental setup for detecting gas at an elevated height. (**a**) Top view; (**b**) side view. The photo-ionization gas detector (PID) reading measured when quadcopter 1 was hovering was compared to that measured when landed.

The setup of the experiment conducted to see the effect of the fountain flow is shown in Figure 5. Saturated ethanol vapor was released from the source fixed on the floor, and the PID was placed at a height of 0.4 m over the gas source. At the start of the experiment, quadcopter 1 was landed on the floor, whereas quadcopter 2 was made to hover at 0.5 m throughout the experiment. The gas release was started 1 min after the start of the experiment, and at the same time, quadcopter 1 was made to hover at the same height as quadcopter 2. After 2 min of hovering, quadcopter 1 was brought back to the floor and kept landed for 2 min. This cycle was repeated twice to check the reproducibility of the result. The polypropylene string connecting the quadcopters was removed in this experiment to avoid the PID from being tangled.

**Figure 6:**
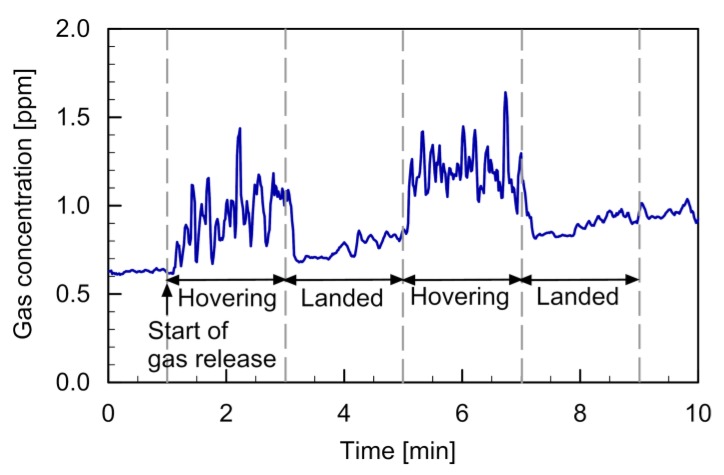
Time course of the PID reading recorded in the gas detection experiment.

The PID reading observed in this experiment is shown in Figure 6. Gas release was started 1 min after the start of recording the PID reading. At the same time, quadcopter 1 was made to hover from the floor. Then, the fountain flow was generated, as shown in Figure 1b and Figure 3b. Ethanol vapor released from the gas source was brought up to the measurement point, and the value of the gas concentration measured by the PID showed a significant increase (1–3 min). When quadcopter 1 was landed, the ethanol vapor released from the source was blown to the left side of Figure 5b by the wall jet generated by quadcopter 2. Therefore, almost no gas was detected by the PID in this period (3–5 min). Similar changes in the PID reading were observed for the other cycle of hovering/landing (5–9 min), although the background ethanol vapor concentration gradually increased over time owing to the continuous release of ethanol vapor in the closed room.

The result shown in Figure 6 indicates that the gas released from the ground can be detected at an elevated height by generating the fountain flow. The large concentration fluctuations observed during the hovering flight of quadcopter 1 indicates that the fountain flow generated by the two connected quadcopters was highly turbulent. Although saturated ethanol vapor was released from the gas source, the concentration was significantly diluted while being transported for 0.4 m from the floor to the position of the PID. Similar concentration values were observed when the PID was placed 3 m from the gas source in an outdoor environment. Therefore, the dilution factor of gas when transported by the fountain flow for 0.4 m was similar to that when transported by natural wind for 3 m.

### 3.2. Range of Gas Detection

**Figure 7:**
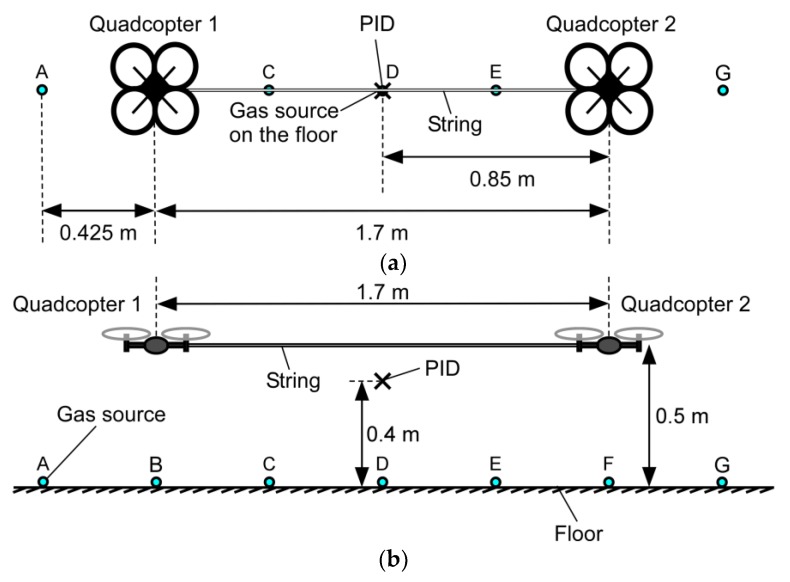
Experimental setup to investigate the range of gas detection along the two quadcopters. (**a**) Top view; (**b**) side view. The position of the gas source was moved from point A through points B–F to point G.

Figure 7 shows the setup of the experiment conducted to investigate the range of gas detection along the two quadcopters. In this experiment, the tube from which ethanol vapor was released was fixed at the tip of a rod, and the position of the gas release was moved from point A through points B–F to point G every 1.5 min. As in the experiment described in the previous section, the two connected quadcopters were made to hover at 0.5 m from the floor. The PID was placed 0.4 m from the floor in the fountain flow generated by the quadcopters.

**Figure 8:**
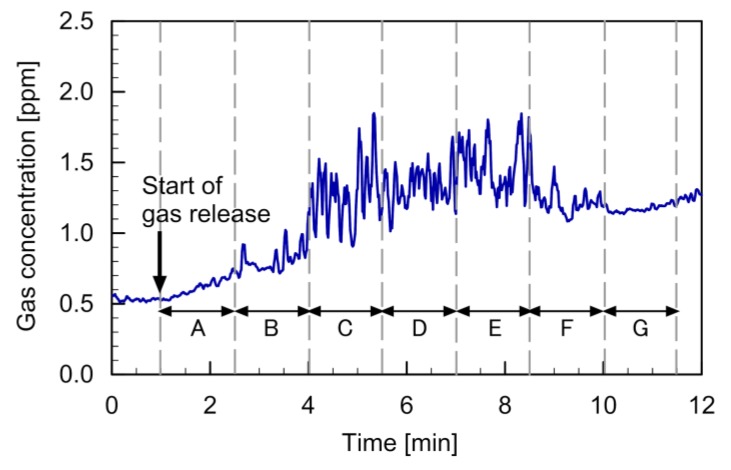
Time course of the PID reading observed when the gas source was moved from point A to point G.

The PID reading observed in this experiment is shown in Figure 8. The gas release from point A was started 1 min after the start of recording the PID reading. However, gas released at this position was blown away from the PID by the leftward wall jet generated by quadcopter 1. The gradual increase in the measured concentration in this period was caused by the gradual increase in the background gas concentration in the room. When the gas source was moved to point B, several peaks appeared in the time course of the PID reading (2.5–4 min). This indicates that a part of ethanol vapor released at point B was brought to the position of the PID by the airflow generated by the quadcopters. A larger amount of gas was transported to the PID when the gas source was moved to points C–E (4–8.5 min). Some gas was detected when the gas source was moved to point F (8.5–10 min), which was directly under quadcopter 2. However, almost no gas was detected when the gas source was moved to point G (10–11.5 min). The result shows that gas puffs released from anywhere between points B and F can be detected if a gas sensor is attached at the midpoint of the string of the two connected quadcopters.

**Figure 9:**
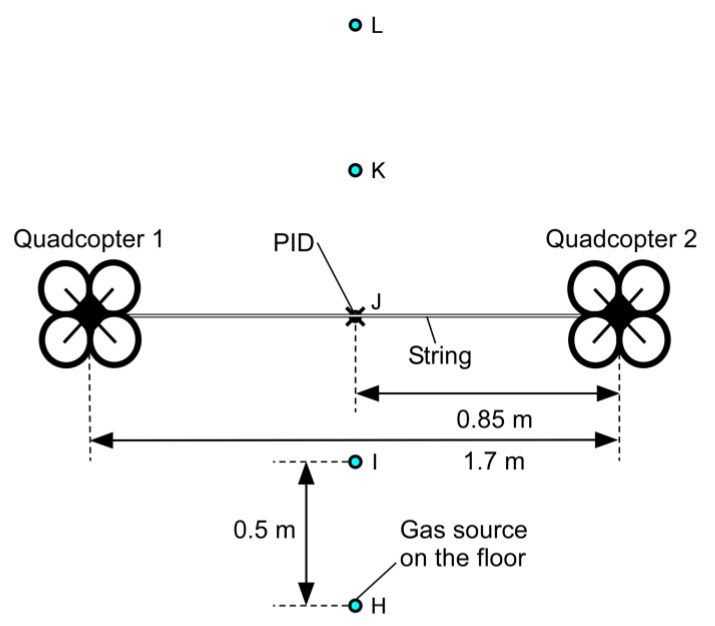
Top view of the experimental setup to investigate the range of gas detection in the direction perpendicular to the two quadcopters. The position of the gas source was moved from point H through points I–K to point L.

**Figure 10:**
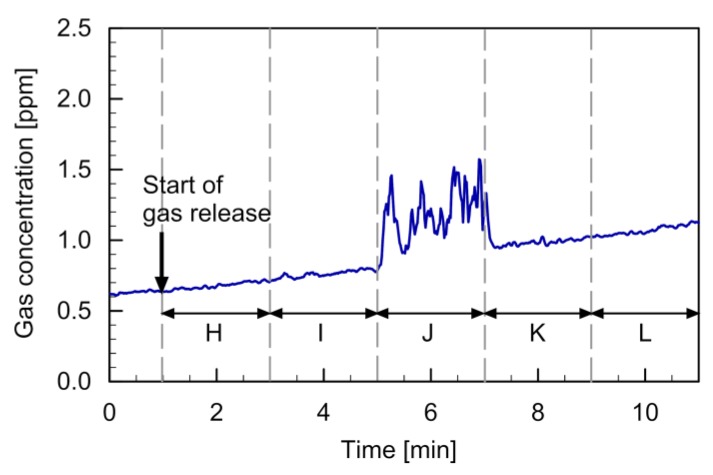
Time course of the PID reading observed when the gas source was moved from point H to point L.

Figure 9 shows the setup of the experiment conducted to investigate the range of gas detection in the direction perpendicular to the two quadcopters. The position of the gas source was moved from point H through points I–K to point L every 2 min. The PID reading observed in this experiment is shown in Figure 10. The gas release was, again, started 1 min after the start of recording the PID reading. The gradual increase in the background gas concentration in the room was observed as in the other experiments. The response to the gas transported by the fountain flow was observed only when the gas source was placed directly under the gas sensor (5–7 min). The result shows that almost no gas can be detected when the gas source is moved slightly off to the side from the line segment between the two quadcopters.

## 4. Results of Flight Experiments

### 4.1. Experiment without Cross-Flow

**Figure 11:**
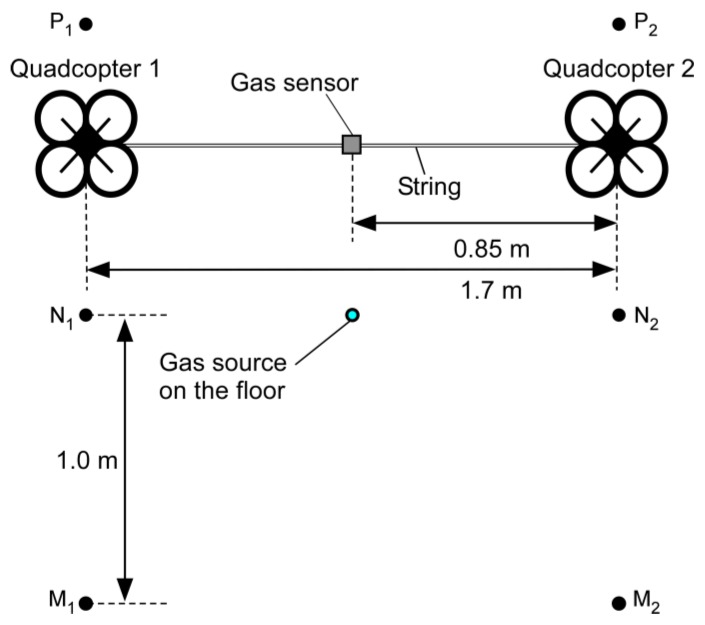
Setup of the flight experiment under no cross-flow conditions (top view). Quadcopter 1 was moved from point M_1_ through point N_1_ to point P_1_, while quadcopter 2 was moved from point M_2_ through point N_2_ to point P_2_.

The experimental results presented in Section 3 showed that gas puffs wafting near the floor can be transported to an elevated height using the fountain flow generated by the two connected quadcopters. Therefore, we proceeded to flight experiments with a MiCS-5521 metal oxide gas sensor attached on the string. Figure 11 shows the setup of the flight experiments without cross-flow. Again, the experiments were conducted in the closed room with all air-conditioning and ventilation systems turned off. Saturated ethanol vapor was released at a constant flow rate of 0.5 L/min from the gas source placed on the floor. Quadcopters 1 and 2 were first made to hover at points M_1_ and M_2_, respectively. Then, they were moved to points N_1_ and N_2_ and made to hover for 1 min. Finally, the quadcopters were moved to points P_1_ and P_2_. The hovering height was kept at 0.5 m throughout the flight.

**Figure 12:**
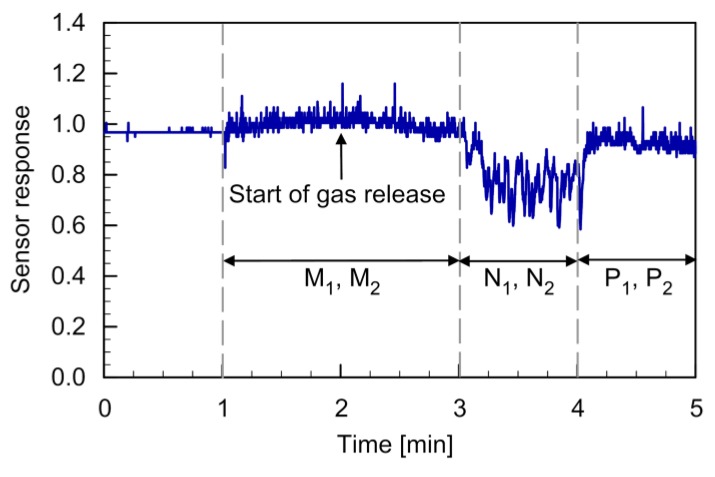
Response curve of the MiCS-5521 sensor during the flight under no cross-flow conditions. Reprinted from [24].

Figure 12 shows the response curve of the MiCS-5521 sensor observed during the flight. The two quadcopters were landed on the floor when the recording of the sensor response was started. At 1 min from the start of recording, the quadcopters were made to hover at 0.5 m at points M_1_ and M_2_. When the quadcopters started to generate the downwashes, the response value of the gas sensor started to show random fluctuations because the sensor was exposed to the turbulent fountain flow. Gas release was started 1 min after the start of hovering. However, almost no change was observed in the gas sensor response while hovering at points M_1_ and M_2_ (2–3 min). When the quadcopters were moved to points N_1_ and N_2_, a significant decrease in the sensor response value was observed (3–4 min). When the quadcopters were hovering at these points, the gas sensor was located immediately above the gas source. The gas released from the source was transported by the fountain flow to the gas sensor onboard the quadcopters. When the quadcopters were moved to points P_1_ and P_2_, the sensor response returned to the baseline level (4–5 min). The reproducibility of the result was confirmed by the triplicated experiments.

The gas sensor response shown in Figure 12 and the PID readings in Figure 6 were measured under similar experimental conditions. It can be seen by comparing these figures that the sensor response value of 0.8 corresponds to the ethanol concentration of 1–1.5 ppm. The response time of a metal oxide semiconductor gas sensor when exposed to gas is typically less than a few seconds, although it generally takes more than 30 s for the response to recover after removal of the gas [27]. In Figure 12, there was a delay of approximately 10 s before the sensor response value reached the new value when the quadcopters were moved to points N_1_ and N_2_. It took several seconds to move the quadcopters manually from points M_1_ and M_2_ to points N_1_ and N_2_, respectively. Then, it took another several seconds for the fountain flow to be fully developed at these new points. Future work should be addressed to investigate the ways to reduce the time to establish the fountain flow.

### 4.2. Experiment with Cross-Flow

**Figure 13:**
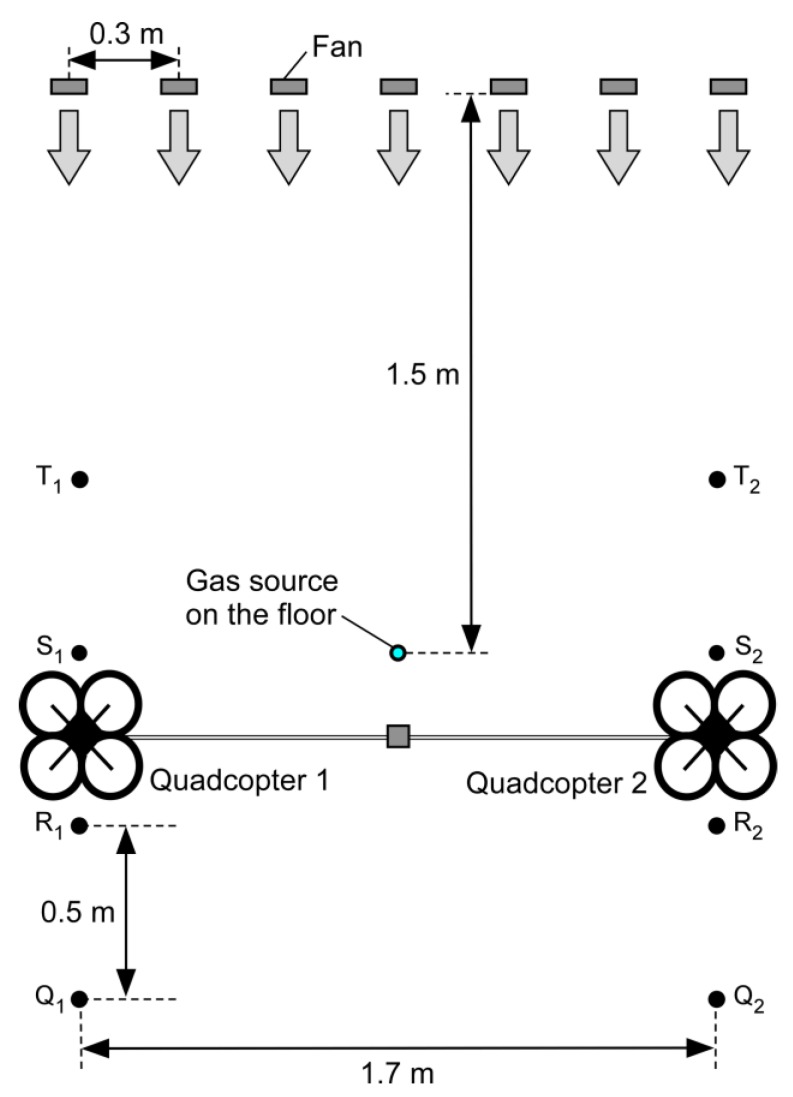
Setup of the flight experiment under cross-flow conditions (top view). Seven electric fans were aligned to generate a uniform airflow field. Quadcopter 1 was moved from point Q_1_ through points R_1_ and S_1_ to point T_1_, while quadcopter 2 was moved from point Q_2_ through points R_2_ and S_2_ to point T_2_.

Figure 13 shows the setup of the flight experiments with a cross-flow. Seven 24 W electric fans (San Ace 9GA0912P1H03, Sanyo Denki Co., Ltd., Tokyo, Japan) were aligned with a spacing of 0.3 m on 28 cm high cardboard boxes. When the fans were activated, a uniform cross-flow of 2 m/s was generated in this field. The gas source was placed 1.5 m from the fans. Saturated ethanol vapor was released at a constant flow rate of 0.5 L/min. Quadcopters 1 and 2 were first made to hover at points Q_1_ and Q_2_, respectively. Then, they were moved to points R_1_ and R_2_. After hovering for 0.5 min, the quadcopters were moved to points S_1_ and S_2_ and made to stay there for 0.5 min. Finally, the quadcopters were moved to points T_1_ and T_2_. The hovering height was kept at 0.5 m throughout the flight.

**Figure 14:**
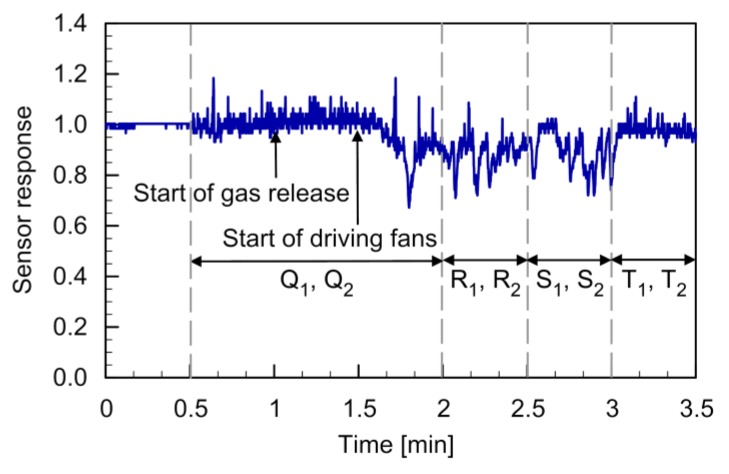
Response curve of the MiCS-5521 sensor during the flight under cross-flow conditions.

The response curve of the MiCS-5521 sensor observed during the flight is shown in Figure 14. The two quadcopters were landed on the floor when the recording of the sensor response was started. At 0.5 min from the start of recording, the quadcopters were made to hover at 0.5 m at points Q_1_ and Q_2_. No significant change in the level of the sensor response was observed, although the random fluctuations of the sensor response increased (0.5–1 min). Gas release was started at 1 min, but the sensor still showed no response change (1–1.5 min). The fans for the cross-flow generation were turned off at that time. Therefore, the released gas remained in the vicinity of the gas source. When the fans were turned on at 1.5 min, the response value of the sensor started to decrease (1.5–2 min). The gas released from the source was first transported by the cross-flow generated by the fans to the area under the two quadcopters. Then, the gas was mixed into the fountain flow and brought to the gas sensor on the string. The response value of the gas sensor remained at the low level when the quadcopters were moved to the next two sets of points (2–3 min). When the quadcopters were moved to points T_1_ and T_2_, the gas sensor response recovered (3–3.5 min) because there was almost no gas on the upwind side of the gas source.

The result shown in Figure 14 indicates that gas puffs wafting near the floor can be detected even if there is a moderate cross-flow. The velocity of the cross-flow was comparable to the velocity of the upward fountain flow shown in Figure 3b. Therefore, the formation of the fountain flow must have been affected by the cross-flow. Nevertheless, the gas was transported from the floor to the sensor on the string. Steel’s test showed that the time-averaged sensor response values when the quadcopters were hovering at the first (Q_1_ and Q_2_, 1.5–2 min), second (R_1_ and R_2_, 2–2.5 min), and third locations (S_1_ and S_2_, 2.5–3 min) were all significantly smaller than the average sensor response value before the gas release (0.5–1 min). The reproducibility of the result was confirmed by duplicated experiments. The gas released from the source on the floor was detected not only when the quadcopters were hovering immediately over the gas source location but also when hovering at positions downwind from the gas source.

This feature can be both advantageous and disadvantageous. If the gas is detected only when the quadcopters are hovering exactly above the gas source location, it would be difficult to find a small gas source in a large field. If the sensor shows response not only at the source location but also at its downstream locations, the chances of detecting the gas are higher even when the field is coarsely scanned. However, it becomes difficult to pinpoint the location of the gas source. At 1.5 min in Figure 14, the velocity of the cross-flow changed from 0 to 2 m/s by turning on the fans. Then, the cross-flow brought the gas to the area under the quadcopters, and the gas sensor started to show a response with some delay. It can be expected from this result that the sensor response will fluctuate if the cross-flow velocity is varied randomly as in real environments. Even a stationary sensor or a sensor placed on a ground robot will show fluctuations in variable winds because the amount of gas transported to the sensor fluctuates. The sensor on the connected quadcopters will show even larger fluctuations because the fountain flow that brings gas from the ground to the quadcopters also fluctuates by the wind variations. Future work should be addressed to quantify the effect of the wind variations and devise signal-processing methods for working around this issue.

## 5. Conclusions

In this paper, we proposed a new method to accomplish gas sensing on multicopters. A multicopter generates strong downwash during flight. Gas puffs released from a gas source on the ground are blown away by this downwash when a multicopter is flying at a low altitude over a gas source. However, if two multicopters are connected, two downwashes impinging on the ground create upward fountain flow in the middle. We showed that this fountain flow can be exploited to transport gas puffs wafting near the ground surface to a gas sensor on the multicopters. For the two connected quadcopters with a frame size of 40 cm, the velocity of the upward fountain flow was approximately 2 m/s. In the experiments, ethanol vapor released from the gas source on the floor was successfully detected by the onboard gas sensor even if there was a cross-flow with a velocity similar to that of the fountain flow.

One of the practical scenarios in which the proposed connected quadcopter system can be used will be methane emission monitoring in landfill sites. Methane has a strong greenhouse effect, and waste landfill is recognized as a major contributor of anthropogenic methane emission [4,5]. To obtain an accurate estimate of the total methane emission from a landfill site is of great importance for assessing its impact on the global climate. Such landfill sites are often covered flat with a layer of soil. Generally, sparsely distributed hotspots (high emission areas) first need to be identified so that the locations to conduct detailed measurements of methane flux from the soil can be determined. The connected quadcopters will be suitable for scanning the wide-open and flat surface of a landfill site to identify the hotspots.

When two quadcopters are flying in close proximity to each other, their flights become unstable because the air currents produced by each quadcopter interfere with the flight of the other. This problem can be solved by designing a new flight controller. Two quadcopters connected by a rigid rod can be regarded as an octocopter with a unique rotor arrangement. It should not be difficult to design a flight controller circuit board to control the eight rotors to attain stable flight of the octocopter. In our experiments, the gas sensor was exposed to an air current generated by a fan (5 m/s) and a fountain flow generated by the quadcopters (2 m/s). The resistance of the sensor we chose (MiCS-5521) showed no significant rise or drop, although high-frequency fluctuations appeared when exposed to the turbulent flows. If the sensor is exposed to higher airflow velocities or more intense turbulence, we may need to consider placing the sensor in a protection tube as described in Reference [14].

A major drawback of the proposed method is that the gas puffs are diluted while being transported from the ground surface to the gas sensor. However, despite the strong turbulence of the fountain flow, the dilution was not as significant as we had anticipated. The dilution factor when transporting gas puffs over 0.4 m by the fountain flow was equivalent to that when transporting gas puffs over 3 m by natural wind. We believe that this dilution factor is acceptable in various gas-sensing applications. To reduce the dilution factor, a faster but less turbulent fountain flow needs to be generated by optimizing the arrangement of the rotors. Currently, the arrangement of rotors in multirotor drones is optimized with respect to their flight performance. However, different results may be obtained if the rotor arrangement is optimized with respect to the efficiency in gas sensing.

Another drawback is the difficulty of achieving quantitative measurement of the gas concentration. We have confirmed that fountain flow is generated even when balls with diameters of 65 and 80 mm are distributed over the floor. However, the uneven surface introduces eddies in the fountain flow. Therefore, the gas puffs become more diluted while being transported from the floor to the gas sensor. It is difficult to predict the concentration of the gas drifting near the floor from the observed gas sensor response without knowing the conditions of the floor surface. Nevertheless, we believe that the proposed system is of some practical use. In methane emission monitoring, for example, the connected drones can be used to distinguish the areas with high methane emission and those with no emission. In some gas source localization algorithms [14,28], the response values of gas sensors are thresholded into binary values, and, yet, the location of a gas source can be successfully estimated using the binary gas information. The connected drones can be used with such algorithms.

It should also be noted that ethanol vapor is heavier than air and tends to stay on the floor. When the detection target is changed to gas with lower density, e.g., methane emitted in a landfill site, detection on multicopters becomes slightly easier. Although the difference in the density between the target gas and air is almost negligible for the concentration ranges observed in the experiments (<2 ppm), future work should include experiments with lighter gases including water vapor and methane. Future work should also include testing the proposed systems in an outdoor field with some roughness on the ground surface.

## References

[1] Kowadlo G., Russell R.A. (2008). Robot odor localization: A taxonomy and survey. Int. J. Robot. Res..

[2] Ishida H., Wada Y., Matsukura H. (2012). Chemical sensing in robotic applications: A review. IEEE Sens. J..

[3] Hernandez Bennetts V., Lilienthal A.J., Neumann P.P., Trincavelli M. (2012). Mobile robots for localizing gas emission sources on landfill sites: Is bio-inspiration the way to go?. Front. Neuroeng..

[4] Abichou T., Chanton J., Powelson D., Fleiger J., Escoriaza S., Lei Y., Stern J. (2006). Methane flux and oxidation at two types of intermediate landfill covers. Waste Manag..

[5] Ishigaki T., Yamada M., Nagamori M., Ono Y., Inoue Y. (2005). Estimation of methane emission from whole waste landfill site using correlation between flux and ground temperature. Environ. Geol..

[6] Bamberger R.J., Watson D.P., Scheidt D.H., Moore K.L. (2006). Flight demonstrations of unmanned aerial vehicle swarming concepts. Johns Hopkins APL Tech. Dig..

[7] Badia S.B., Bernardet U., Guanella A., Pyk P., Verschure P.F.M.J. (2007). A biologically based chemo-sensing UAV for humanitarian demining. Int. J. Adv. Robot. Syst..

[8] Ishida H. (2009). Blimp robot for three-dimensional gas distribution mapping in indoor environment. AIP Conf. Proc..

[9] Neumann P.P., Asadi S., Lilienthal A.J., Bartholmai M., Schiller J.H. (2012). Autonomous gas-sensitive microdrone: Wind vector estimation and gas distribution mapping. IEEE Robot. Autom. Mag..

[10] Rossi M., Brunelli D. (2016). Autonomous gas detection and mapping with unmanned aerial vehicles. IEEE Trans. Instrum. Meas..

[11] Frish M.B., Wainner R.T., Green B.D., Laderer M.C., Allen M.G. (2005). Standoff gas leak detectors based on tunable diode laser absorption spectroscopy. Proc. SPIE.

[12] Emran B.J., Tannant D.D., Najjaran H. (2017). Low-altitude aerial methane concentration mapping. Remote Sens..

[13] Neumann P.P., Kohlhoff H., Hüllmann D., Lilienthal A.J., Kluge M. Bringing mobile robot olfaction to the next dimension––UAV-based remote sensing of gas clouds and source localization. Proceedings of the IEEE International Conference on Robotics and Automation.

[14] Neumann P.P., Hernandez Bennetts V., Lilienthal A.J., Bartholmai M., Schiller J.H. (2013). Gas source localization with a micro-drone using bio-inspired and particle filter-based algorithms. Adv. Robot..

[15] Golston L.M., Aubut N.F., Frish M.B., Yang S., Talbot R.W., Gretencord C., McSpiritt J., Zondlo M.A. (2018). Natural gas fugitive leak detection using an unmanned aerial vehicle: Localization and quantification of emission rate. Atmosphere.

[16] Takei Y., Kanazawa Y., Hirasawa K., Nanto H. Development of 3D gas source localization using multi-copter with gas sensor array. Proceedings of the ISOCS/IEEE International Symposium on Olfaction and Electronic Nose.

[17] Burgués J., Hernández V., Lilienthal A.J., Marco S. (2019). Smelling nano aerial vehicle for gas source localization and mapping. Sensors.

[18] Eu K.S., Yap K.M., Tee T.H. (2014). An airflow analysis study of quadrotor based flying sniffer robot. Appl. Mech. Mater..

[19] Luo B., Meng Q.-H., Wang J.-Y., Ma S.-G. (2016). Simulate the aerodynamic olfactory effects of gas-sensitive UAVs: A numerical model and its parallel implementation. Adv. Eng. Softw..

[20] Dewan A., Dutta R., Srinivasan B. (2012). Recent trends in computation of turbulent jet impingement heat transfer. Heat Transf. Eng..

[21] Abdel-Fattah A. (2007). Numerical and experimental study of turbulent impinging twin-jet flow. Exp. Therm. Fluid Sci..

[22] Li Q., Page G.J., McGuirk J.J. (2007). Large-eddy simulation of twin impinging jets in cross-flow. Aeronaut. J..

[23] Weigand B., Spring S. (2011). Multiple jet impingement––A review. Heat Transf. Res..

[24] Tanaka K., Koguchi S., Sato R., Ramirez J.P.R., Matsukura H., Ishida H. Using airflows generated by multicopter for gas detection in midair. Proceedings of the 17th International Meeting on Chemical Sensors.

[25] Zhang J., Liu X., Neri G., Pinna N. (2016). Nanostructured materials for room-temperature gas sensors. Adv. Mater..

[26] Hsi P.C. (1998). Photo-Ionization Detector for Volatile Gas Measurement. U.S. Patent.

[27] Yamanaka T., Ishida H., Nakamoto T., Moriizumi T. (1998). Analysis of gas sensor transient response by visualizing instantaneous gas concentration using smoke. Sens. Actuators A Phys..

[28] Li J.-G., Meng Q.-H., Wang Y., Zeng M. (2011). Odor source localization using a mobile robot in outdoor airflow environments with a particle filter algorithm. Auton. Robots.

